# Effect of acute iron infusion on insulin secretion: A randomized, double-blind, placebo-controlled trial

**DOI:** 10.1016/j.eclinm.2022.101434

**Published:** 2022-05-06

**Authors:** Evrim Jaccard, Kévin Seyssel, Alexandre Gouveia, Catherine Vergely, Laila Baratali, Cédric Gubelmann, Marc Froissart, Bernard Favrat, Pedro Marques-Vidal, Luc Tappy, Gérard Waeber

**Affiliations:** a Department of Medicine, Lausanne University Hospital (CHUV) and University of Lausanne, rue du Bugnon 46, Lausanne 1011, Switzerland; b Department of Biomedical Sciences, Faculty of Biology and Medicine, University of Lausanne, rue du Bugnon 7a, Lausanne 1005, Switzerland; c Center for Primary Care and Public Health, University of Lausanne, rue du Bugnon 44, Lausanne, Switzerland; d Clinical Research Center, CHUV, University of Lausanne, Switzerland; e Pathophysiology and Epidemiology of Cerebro-Cardiovascular Diseases (PEC2, EA7460),UFR des Sciences de Santé, University of Bourgogne Franche-Comté, 7 boulevard Jeanne d’ Arc, Dijon 21079, France

**Keywords:** Type 2 diabetes, Insulin secretion, Insulin sensitivity, Iron deficiency, Iron sufficiency, Inflammation

## Abstract

**Background:**

Chronic exposure to high iron levels increases diabetes risk partly by inducing oxidative stress, but the consequences of acute iron administration on beta cells are unknown. We tested whether the acute administration of iron for the correction of iron deficiency influenced insulin secretion and the production of reactive oxygen species.

**Methods:**

Single-center, double-blinded, randomized controlled trial conducted between June 2017 and March 2020. 32 women aged 18 to 47 years, displaying symptomatic iron deficiency without anaemia, were recruited from a community setting and randomly allocated (1:1) to a single infusion of 1000 mg intravenous ferric carboxymaltose (iron) or saline (placebo). The primary outcome was the between group mean difference from baseline to day 28 in first and second phase insulin secretion, assessed by a two-step hyperglycaemic clamp. All analyses were performed by intention to treat. This trial was registered in ClinicalTrials.gov NCT03191201.

**Findings:**

Iron infusion did not affect first and second phase insulin release. For first phase, the between group mean difference from baseline to day 28 was 0 μU × 10 min/mL [95% CI, -22 to 22, *P* = 0.99]. For second phase, it was -5 μUx10min/mL [95% CI, -161 to 151; *P* = 0.95] at the first plateau of the clamp and -249 μUx10min/mL [95% CI, -635 to 137; *P* = 0.20] at the second plateau. Iron infusion increased serum ascorbyl/ascorbate ratio, a marker of plasma oxidative stress, at day 14, with restoration of normal ratio at day 28 relative to placebo. Finally, high-sensitive C-reactive protein levels remained similar among groups.

**Interpretation:**

In iron deficient women without anaemia, intravenous administration of 1000 mg of iron in a single sitting did not impair glucose-induced insulin secretion despite a transient increase in the levels of circulating reactive oxygen species.

**Funding:**

The Swiss National Science Foundation, University of Lausanne and Leenaards, Raymond-Berger and Placide Nicod Foundations.

**Research in context**

**Evidence before this study**

On October first, 2021, we searched PubMed with no time and language restrictions, for publications on iron's impact on diabetes risk using the following search terms: “iron”, “ferritin”, “glucose homeostasis”, “insulin resistance” and “type 2 diabetes”.

Individuals with pathologic iron overload such as those suffering from haemochromatosis (HH) or displaying transfusional iron overload in the context of beta thalassaemia major have an increased incidence of type 2 diabetes (T2D). The impact that high body iron stores have on glucose homeostasis and T2D risk has been mostly studied through epidemiological surveys or after lowering iron stores towards target levels by phlebotomies. The later approach provided inconsistent results and is subject to bias as phlebotomies induce a transient hypoxemic environment known to cause hypoxia inducible factor stabilisation, which could mediate the effects attributed to the lowering of iron stores.

**Added value of this study**

This study was designed to measure acute changes in glucose homeostasis induced by an acute IV injection of iron. Iron-deficient non-anaemic symptomatic women either intolerant or unresponsive to oral iron formulations received either 1000 mg of ferric carboxymaltose or saline placebo infusion. Our aim was to unveil iron's toxic effects on beta cell function. We hypothesized that iron would impair beta cell homeostasis and that it could be measured by a two-step hyperglycaemic clamp as a functional decrease in first and second-phase insulin secretion at 28 days. We also hypothesized that this decrease in beta cell function would coincide with increased reactive oxygen species production. We found that despite ferritin levels remaining in the “overload range” (i.e. Ferritin > 200 ng/ml) during the whole study period, there was no statistical difference in mean values for first and second phase insulin secretion, although a transient increase in the levels of reactive oxygen species was measured.

**Implications of all evidence available**

Our results show that an acute infusion of 1000 mg intravenous iron to apparently healthy but iron deficient women did not alter insulin secretion at 28 days, despite a transient increase in the levels of circulating reactive oxygen species. The limitations of this study are the small sample size, the lack of previous trials to inform sample size calculation and the incomplete reproduction of the time-dose exposure linked to chronic iron exposure. All these factors could have lead to the null hypothesis.

Alt-text: Unlabelled box

## Introduction

The role of excess body iron in the pathogenesis of type 2 diabetes (T2D) has been the subject of intense research.^1^, ^2^, ^3^, ^4^ Several diseases leading to systemic iron overload such as Hereditary Haemochromatosis (HH) or thalassaemia major are associated with an increased incidence of T2D. However, the relative contribution of genetic factors to iron overload and the relative risk of diabetes imputable to iron compared to traditional risk factors of T2D remains unclear.^2^^,^^5^, ^6^, ^7^ Nevertheless, our knowledge about the relationship between body iron stores and glucose homeostasis is mostly derived from epidemiological surveys^8^^,^^9^ or interventional studies aiming at lowering iron stores towards target levels.^10^, ^11^, ^12^, ^13^, ^14^, ^15^ The latter was achieved through phlebotomies and led to conflicting results, some reports showing an improvement in glucose homeostasis in patients with HH,^11^ T2D,^12^ glucose intolerance,^14^ metabolic syndrome (MetS)^15^ or in healthy blood donors,^10^^,^^13^ while others showed no improvement in patients with HH^16^ or MetS.^17^ Besides, several systematic reviews with or without meta-analysis showed no conclusive evidence for significant improvement of metabolic parameters after phlebotomy.^16^^,^^18^, ^19^, ^20^ Importantly, interventions relying on phlebotomy decrease iron stores but also induce a transient hypoxemic environment.^16^^,^^21^ In this regard, both iron deficiency and hypoxia are synergistic stabilizers of hypoxia-inducible factors (HIFs), whose activation has been shown to improve glucose homeostasis.^22^^,^^23^ Therefore, the link between phlebotomies, a putative improvement in glucose homeostasis and iron is elusive as other factors than iron could explain this link.

Contrary to chronic iron overload, it is unknown whether a quick modification of iron stores, such as that occurring after a single acute intravenous (IV) iron infusion, changes insulin secretion or the production of reactive oxygen species (ROS). The latter is generated when iron is present in excess and to which insulin secreting beta cells are exquisitely sensitive.^1^^,^^2^^,^^4^

Hence, we aimed to assess if acute iron infusion affects insulin secretion or ROS production by conducting a double-blind, placebo-controlled trial. We compared the effect of an IV administration of a single infusion of 1000 mg of ferric carboxymaltose (FCM) relative to placebo. Our hypothesis was that in this setting, iron would impair beta cell homeostasis and that it could be measured by a two-step hyperglycaemic clamp as a functional decrease in first and second-phase insulin secretion. We also hypothesized that this decrease in beta cell function would coincide with increased ROS production. Such data are important to gather as IV iron is frequently administered in primary care, often to fatigued iron deficient women, sometimes out of convenience, although not all safety issues linked to these administrations have been addressed.

## Methods

### Study design

The DIAFER study was an investigator-led, randomized, double-blind, parallel-group, placebo-controlled, proof-of-concept trial conducted at the Lausanne University Hospital, Switzerland, from June 21, 2017 to March 9, 2020. The Clinical Research center monitored the trial and ensured compliance with the International Conference on Harmonization guidelines for Good Clinical Practice (ICH E6(R2)) and the Declaration of Helsinki. The trial was registered at ClinicalTrials.gov (NCT03191201) and approved by the regional ethics committee (2016-01449). No changes were made to eligibility criteria and design after the trial started. In the original protocol of this study, insulin secretion, insulin sensitivity, and endogenous glucose production were listed as co-primary outcomes although our primary hypothesis was that iron would impair glucose-induced insulin secretion. This is also the reason why a two-step hyperglycaemic clamp was performed. As we could not discard the hypothesis that iron infusion could alter insulin resistance or endogenous glucose production we therefore also measured these parameters in our protocol. These results are however not reported here for the purpose of simplicity and will be added to a separate report as secondary outcomes of this study. The trial was terminated early after the recruitment of 32 out of 38 planned participants, because of the COVID-19 pandemic.

The study involved a randomized part comprising six visits and two hyperglycaemic clamps and an optional extension arm proposed to placebo arm participants with an additional hyperglycaemic clamp (See Fig. S1 in the Supplementary Appendix). The study involved six visits, numbered V1 to V6, plus an optional seventh visit (V7). V1 was the screening visit. V2 was performed within 12 weeks (average three) of V1 and included the baseline hyperglycaemic clamp. V3 was performed 7 days after V2 and included the administration of the study drug or the placebo. V4 was an interim visit performed 14 ± 2 days after V3. V5 was performed 28 ± 2 days after V3 and included the primary outcome assessment hyperglycaemic clamp. V6 was scheduled at the end of the trial, to administer the study drug to the participants of the placebo group at V3. The optional seventh visit (V7) was proposed to the participants of the placebo group 28 ± 2 days after V6 and included an evaluation hyperglycaemic clamp (Fig. S1).

### Participants

Out of 32 participants, 24 (75%) were recruited through advertisements posted at the University of Lausanne and at the Lausanne University Hospital and 8 (25%) were identified in the pool of patients addressed by their general practitioner to the outpatient clinic of the centre for Primary Care and Public Health (Unisanté) to delegate iron perfusion. All participants were from a community setting and provided written informed consent before entering the study.

Eligible participants were: (1) women aged ≥ 18 years; (2) with symptomatic fatigue (≥ 4 points on a visual analogic scale); (3) reporting intolerance or a lack of response to oral iron formulations; (4) with documented iron deficiency at screening that was defined as serum ferritin comprised between 30 and 50 mg/L if transferrin saturation was < 20% or as serum ferritin < 30 mg/L; (5) with folate and vitamin B12 levels ≥ lower limit of the reference range (to exclude other causes of fatigue); (6) with C-reactive protein levels < 20 mg/L if on oral hormonal contraception , and < 5 mg/L if not on oral hormonal contraception (to exclude individuals with inflammation); (7) with a regular menstrual cycle; (8) with a negative pregnancy test.

Exclusion criteria were: (1) anaemia defined as haemoglobin (Hb) < 117 g/L; (2) a suspicion of a major depressive disorder based on the Patient Health Questionnaire 2 (PHQ-2), a PHQ-2 score > 3 being considered as positive; (3) a self-declared irregular menstrual cycle whose length would reasonably not allow two hyperglycaemic clamps from being scheduled approximately 28 days apart, during the first ten days of the menstrual cycle (potential impact of the menstrual cycle on the results of clamp studies); (4) diabetes, defined as a HbA_1c_ ≥ 6.5% and/or with fasting blood glucose levels ≥ 7 mmol/L and/or history of diabetes and/or use of anti-diabetic drugs; (5) a BMI < 18.5 kg/m^2^ or > 30.0 kg/m^2^; (6) a thyroid stimulating hormone levels < 0.2 or > 10.0 mUI/L; (7) a history of acquired iron overload or HH; (8) a chronic inflammatory disease or an active infection; (9) any malignancy; (10) HIV/AIDS; (11) significantly impaired liver/renal function (transaminases > three times the upper limit of the reference range or estimated glomerular filtration rate by the CKD-EPI equation < 60 mL/min/1.73 m^2^); (12) sleep apnoea; (13) allergic disorders (i.e. asthma or anaphylactic reactions); (14) prior hypersensitivity to i.v. iron drugs or their excipients; (15) the use of iron preparations within four weeks of screening; (16) blood donation in the previous 12 weeks; (17) any side effect imputable to iron that in the opinion of unblinded research staff could unmask group assignment.

Participants were asked at each study visit about the intake of any kind of medication or herbal medicine. The consumption of drugs with anti-inflammatory properties (systemic non-steroidal anti-inflammatory drugs, selective COX-2 inhibitors and systemic corticosteroids) for whatever reason was discouraged and the use of paracetamol was encouraged instead. A list of medication taken by the participant was established at the screening visit and updated at each subsequent visit. No participant was taking any drugs with anti-inflammatory properties at study entry, as this was an implicit exclusion criterion. Participants were asked to contact unblinded study staff in case such medication was considered for use during the study period. Unblinded study staff asked at each visit for the consumption of any kind of medication and reminded the list of prohibited medication such as iron substitutes and drugs with anti-inflammatory properties. No consumption of such drugs was reported during the study.

### Randomization and masking

The trial pharmacist used a web-based automated system to generate the randomization sequence using a 1:1 ratio and block length of two. It was provided using sequentially numbered, sealed, opaque envelopes. Envelopes were matched to participants in ascending order, the next participant scheduled for the baseline metabolic investigation being assigned to the next randomization number/envelope available. Allocation concealment was maintained to the investigator until the end of the assessment hyperglycaemic clamp visit, where the envelopes were opened and signed by the participant and the investigator, along with the date and time of opening.

The administration of study drug took place at visit three (V3) (Fig. S1). To achieve masking of the investigators during the randomized part of the study, unblinded research staff collected the allocated infusion set from pharmacy and administered the study drug to the participants. They also performed all planned procedures of visit four (V4), collected clinical data and questionnaires at the beginning of visit five (V5) and had exclusive access to Case Report Forms or laboratory results during that phase. Participants were also instructed to exclusively contact unblinded research staff in case of any change in their health status during the study. Except the measure of weight at V4, unblinded research staffs were otherwise not involved in measuring outcomes and had no contact with the investigators.

To achieve masking of the participant, the infusion bags were covered with non-translucent bags, opaque infusion lines were used and a curtain shielded the infusion arm from the participant. All study staff performing laboratory measurements were blinded to treatment allocation.

### Procedures

FCM (1000 mg iron) was diluted in 250 mL sterile 0.9% sodium chloride solution and administered as a single infusion over 30 min. Placebo-treated participants received 250 mL of saline solution under identical conditions.

### Outcomes

The primary outcome was the average change in first and second phase insulin secretion from baseline to 28 days post treatment and between groups as assessed by a dynamic two-step hyperglycaemic clamp. First phase insulin release, reflecting the early insulin peak secreted from the pancreatic beta cell in response to glucose stimulation , was calculated as the area under the curve minus the baseline value (iAUC) for insulin as per the trapezium method during the first 10 min of the clamp, minus the area under fasting insulin during the same period (μU x 10 min/mL), fasting insulinemia being defined as the insulinemia at the time 0 of the clamp. First phase insulin release was elicited by a 12 mg/kg/min bolus of exogenous glucose 20% (G 20; B. Braun Medical AG) during time 0 to 2 min, followed by a 6 mg/kg/min bolus during time 2 to 4 min, followed by a 3 mg/kg/min bolus during time 4 to 6 min, followed by a 6 mg/kg/min bolus during time 6 to 10 min. Second phase insulin release, reflecting beta cell function under sustained elevated glucose levels, was calculated at each step of hyperglycaemia from the average values of plasma insulin obtained during the second half-hour of each plateau (times 60 to 90 min and 150 to 180 min) minus the area under the fasting insulin curve during the same period (μU x 30 min/mL).

Secondary outcomes were mean changes from baseline to 14 and 28 days post treatment and between groups in: (1) circulating oxidative stress levels, and (2) high-sensitive C-reactive protein (hsCRP) levels. Circulating oxidative stress level was assessed by measuring plasma ascorbate, plasma ascorbyl free radical (which allowed the calculation of the ascorbyl/ascorbate ratio), and the total plasma antioxidant capacity using the oxygen radical absorbance capacity (ORAC) as previously described.^24^ Total ascorbate concentration was measured by high performance liquid chromatography using fluorimetric detection (λexc= 360 nm, λem = 440 nm) and plasma ascorbyl free radical was measured by electron spin resonance (ESR) spectroscopy as previously described.^25^^,^^26^ Samples for the determination of hsCRP were immediately processed following established clinical procedures at the Lausanne University Hospital's laboratory, using the kit N° 11 972 855 216 from Roche Diagnostics.

Each clamp was planned during the presumed first ten days of the menstrual cycle of the participant. The first day of menstruation was defined as day 0 as long as participants were taking either no hormonal contraception or a cycle preserving one. In case they were taking continuous hormonal contraception, they were considered to be in the middle of the first ten days of the interval of interest (i.e. at day five). The start of menstruation was self-declared. Participants were instructed not to do any physical activity the day before the test and not to smoke on the morning of the test. Each clamp was preceded, the evening before, by a standardised meal (i.e. energy requirements calculated with the Harris-Benedict equation and physical activity factor of 1.5) containing 55% carbohydrate (45% complex carbohydrate and 10% sugar), 15% protein and 30% fat. All meals were prepared by our staff and given to participants with instructions relative to the timing of intake. The hyperglycaemic clamp was performed as previously described.^27^^,^^28^ For each test, participants reported to the metabolic investigation laboratory at 6:30 AM after a ten hours overnight fast. On arrival, participants were asked to void, and were then weighed and had their body composition assessed by bio-electrical impedance (Biacorpus RX 400, Medi Cal HealthCare GmbH, Germany). Thereafter, they rested in a semi-recumbent position in bed until completion of the test. An indwelling venous catheter was inserted into an antecubital vein of each forearm. A primed variable infusion of exogenous glucose 20% (G 20; B. Braun Medical AG) was started at time 0 min of the clamp using a Y-connector on one of the forearm catheters. It allowed to increase plasma glucose levels to reach the first plateau at 7.5 mmol/L glucose that was maintained for 60 min (time 30 to 90 min) and then to reach a second plateau at 10 mmol/L glucose that was maintained for an additional 60 min (time 120 to 180 min) (Fig. S2)().^27^^,^^29^ The cannula on the opposite forearm was used for periodic blood sampling. The corresponding hand was maintained in a heating device to achieve partial arterialisation of venous blood. Blood samples were collected at times −150, −30, −15, 0, 2, 4, 6, 8, 10, 15, 60, 90, 150 and 180 min for determination of plasma insulin concentrations. During the −150 to 180 min phase, arterialized venous whole blood was collected at −150, −30, −15, 0, 2, 4, 6, 8, 10 min and every 5 min thereafter for blood glucose determinations using an Accu-Chek® guide glucometer from Roche. Each blood glucose measurement was performed in duplicate and averaged to the nearest 0.05 mmol/L. It allowed adjusting the infusion rate of the exogenous 20% glucose solution to achieve the two stable glucose levels at 7.5 mmol/L (first step/plateau, time 30 to 90 min) and at 10 mmol/L (second step/plateau, time 120 to 180 min) respectively (Fig. S2).

Blood samples were collected in EDTA (insulin), or heparin (ascorbate and ascorbyl radical). Plasma was separated from blood cells immediately after collection by centrifugation during 10 min at 3500 rpm at 4 °C and stored at −20 °C until analysed. Samples for the determination of ascorbyl radical were immediately snap-frozen in liquid nitrogen and then stored at – 80 °C until assayed. Samples for the determination of iron indices were immediately processed following established clinical procedures at the Lausanne University Hospital's laboratory. Plasma insulin concentrations were measured with the use of radioimmunoassay kits (Merck Millipore, Billerica, MA, USA).

At baseline, a clinical history (comprising smoking status and use of concomitant medication), physical examination, visual analogue scale (VAS) of fatigue, PHQ-2 questionnaire, the International Physical Activity Questionnaire (IPAQ), weight, height, waist and hip circumference, blood pressure and blood tests were obtained for each participant. At V5, participants’ detailed dietary information was collected with a semi-quantitative food frequency questionnaire developed and validated on a Swiss population.^30^ Total iron intake was estimated using the French CIQUAL food composition database. Body weight was measured without shoes in light indoor clothes to the nearest 0.1 kg using a calibrated portable digital scale (Beurer living® GS58).

### Power calculation

Our assumption was that iron would impair first and second phase insulin secretion at 28 days. Sample size estimates were hampered by the lack of previous clamp studies in patients with or without iron deficiency. The planned sample size of 38 participants was calculated based on the effect of phlebotomy on two hours plasma insulin and glucose concentrations after a 75-g oral glucose load. The following information was considered: an alpha value of 5%, a power of 80%, a one-sided test benefiting the intervention, a 1:1 placebo to intervention ratio, and ¾ of the effect size reported in,^12^ which was 247 pmol/L for insulin and 1.4 mmol/L for glucose. Calculations for plasma insulin thus used an effect size of 185 pmol/L and a standard deviation (SD) of 158 pmol/L and led to a sample size of 10 per group. Calculations for glucose used a difference of 1.0 mmol/L and a SD of 1.2 mmol/L and led to a sample size of 19 per group. The larger sample size was considered. Calculations were performed using Stata version 14.0 (Stata Corp, College Station, Texas, USA), with the *power twomeans* function.

### Statistics

Data was analysed on an intention-to-treat approach. Continuous data derived from clamp studies at baseline (D_0_) and at day 28 (D_28_) were analysed as follows: first, individual differences D_28_–D_0_ were computed, then compared between groups (iron and placebo) using student's *t*-test. Continuous data collected at D_0_, D_14_ and D_28_ were compared between groups using a mixed model for repeated measures.

The Stata syntax for the mixed model was the following:

mixed *outcome* i.**group** time i.group#c.time || ID: time, reml

Where **group** identifies the group (iron or placebo), time identifies the assessment period, and ID corresponds to the participant's identification code. Serum ascorbate levels, serum ascrobyl/ascorbate ratio and serum antioxidant status were normalized for baseline values.

Missing values were neither replaced nor imputed. In the placebo group, no data was missing for main outcomes. In the iron group data was missing for one, first phase insulin secretion and one, second phase insulin secretion at the first step and in total for 3, s phase insulin secretion at second step.

All analyses were conducted with the STATA 16.1 software (StataCorp, College station, TX, USA).

### Role of the funding source

The funding organisations played no role in the design of study, choice of enrolled participants, review and interpretation of data, preparation of manuscript, final approval of manuscript or decision to submit for publication. The datasets analysed during the current study are available from the corresponding author on reasonable request.

## Results

**Figure 1:**
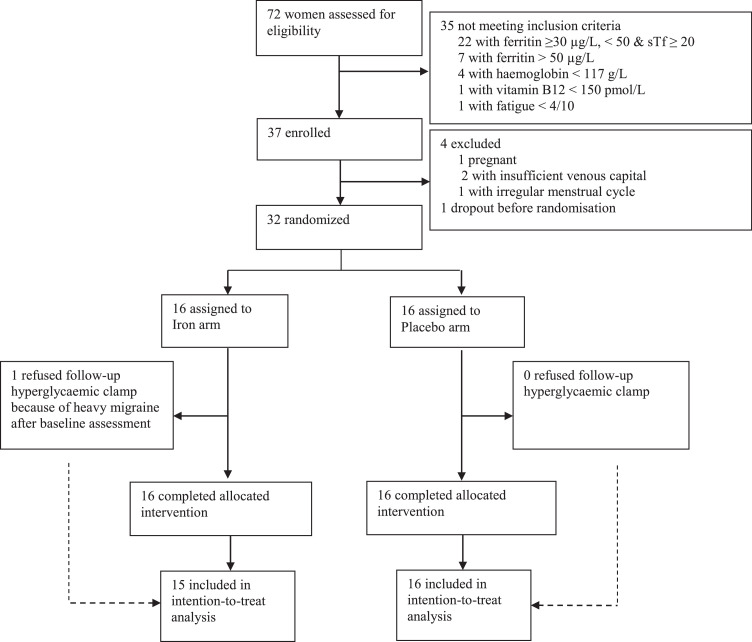
**CONSORT diagram.** Study participation by treatment group. sTf indicated transferrin saturation.

Between May 2017 and March 2020, we screened 72 outpatients for eligibility (Fig. 1) and assigned 32 to either iron (*n* = 16) or placebo (*n* = 16). Three participants of the placebo group took part in the extension arm of the study (Fig. S1). In the iron group, one partial drop-out occurred with no follow-up clamp performed in a participant of the iron arm because of heavy migraine after baseline assessment (Fig. 1), two second steps of the clamp were not performed in two other participants of the iron group, once at the baseline clamp because the venous catheter was accidentally pulled out, preventing the test to resume and once at a follow-up clamp because, a phlebitis arose on the site of the venous catheter were 20% glucose was perfused preventing the second step of the clamp to be performed. The participant of the iron group that refused the follow-up clamp, otherwise accepted all other measurements, blood draws and answered questionnaires.

Besides differences in baseline ferritin levels, there was no further difference between eligible and non-eligible participants (Table S1). There were no differences among groups in demographic, physical and biologic characteristics (Table 1). All participants received their allocated therapy and one participant of the iron group refused the follow-up metabolic investigation (Fig. 1).

**Table 1:** Participants’ characteristics at baseline.

Characteristics	Iron (*n* = 16)	Placebo (*n* = 16)
Age, years	25.6 ± 6.0	30.1 ± 7.9
Ethnicity, *n* (% Caucasian^a^)	12/16 (75)	12/16 (75)
Body weight, kg	62.4 ± 7.5	62.6 ± 7.4
BMI, kg/m^2^	22.6 ± 2.3	22.5 ± 2.5
Heart rate, beats per min	65 ± 8	66 ± 8
Systolic BP, mm Hg	106 ± 7	114 ± 12
Diastolic BP, mm Hg	68 ± 4	74 ± 11
Waist circumference, cm	70.4 ± 5.3	71.1 ± 6.7
Hip circumference, cm	100 ± 7	100 ± 6
Waist-Hip ratio, *n*	0.70 ± 0.05	0.71 ± 0.06
VAS of fatigue, *n*	6.41 ± 1.31	6.28 ± 1.24
PHQ-2, *n*	0.8 ± 1.0	1.5 ± 1.0
Iron perfusions in the past, *n*	1.1 ± 1.8	1.0 ± 1.2
Iron content of the diet, mg/day	22.1 [19.4–26.6]	24.2 [18.5–32.3]
Physical activity level, min/week		
Intense	150 [0 - 180]	45 [0 - 120]
Moderate	120 [95 - 180]	60 [0 - 120]
Walking	160 [120 - 215]	110 [90 - 290]
Sitting	399 [202 - 467]	489 [231 - 561]
Smokers,%	2/16 (12.5)	3/16 (18.8)
Hormonal contraception, *n* (%)	4/16 (25)	4/16 (25)
Biology		
Haemoglobin, g/L	133 ± 10	132 ± 6
MCV, fL	88 ± 4	85 ± 3
Serum ferritin, µg/L	23 ± 8	18 ± 8
Transferrin saturation, *n* (%^b^)	24 ± 9	21 ± 8
C-reactive protein, mg/L	1.3 ± 2.2	1.3 ± 2.4
Venous glycaemia, mmol/L	4.8 ± 0.4	4.8 ± 0.4
HbA_1c_, *n* (%)	5.0 ± 0.2	5.1 ± 0.3
HbA_1c_, mmol/mol	31 ± 2	32 ± 3
Thyroid stimulating hormone, mUI/L	2.4 ± 1.1	2.0 ± 1.2
Vitamin B9, nmol/L	22.3 ± 8.6	14.9 ± 3.6
Vitamin B12, pmol/L	244 ± 88	280 ± 82
Alanine aminotransferase, U/L	16 ± 5	15 ± 3
Aspartate aminotransferase, U/L	18 ± 5	17 ± 3

Data are mean ± SD, numbers (%) or median [interquartile range]. BMI denotes Body Mass Index; BP, Blood Pressure; VAS, Visual Analogue Scale; MCV, Mean Corpuscular Volume; fL, femtoliters and PHQ-2, Patient Health Questionnnaire-2.

a Ethnicity was self-reported.

b The percent transferrin saturation was calculated as iron (in micromoles per liter) ÷ transferrin (in grams per liter) x 25.1.

**Figure 2:**
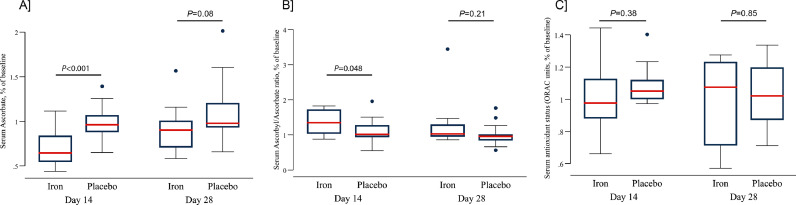
**Iron induced change in the levels of antioxidants and circulating ROS.** Iron transitorily decreased serum ascorbate levels at day 14 with restoration at day 28 (panel A) with no effect for placebo. There was an increase in serum ascrobyl/ascorbate ratio at day 14 with restoration of normal ratio at day 28 and no effect for placebo (panel B). The serum antioxidant status (ORAC units) remained unchanged throughout the study period (panel C). Serum ascorbate levels, serum ascrobyl/ascorbate ratio and serum antioxidant status were normalized for baseline values. The red line in the boxplot is the median of the estimate. *P* values are for the effect of the intervention at 14 and 28 days.

Treatment with iron did not alter insulin secretion as no differences were found between iron and placebo for mean iAUC for first or second phase insulin release (Table 3). An analysis including the participants of the extension arm yielded similar results (Table S3). Iron infusion significantly decreased ascorbate levels and increased the ascorbyl/ascorbate ratio at day 14 with restoration at day 28 (Fig. 2). ORAC values were not different among groups. So was it for hsCRP levels. In both the iron and the placebo groups, 15/16 (94%) of the basal and 14/16 (88%) of the follow-up two-step hyperglycaemic clamps were performed during the first ten days of the menstrual cycle (Table 3). The median iron content of the diet during the study period was comparable in both groups (Table 1).

There was a significant change in the concentration of ferritin and transferrin but not of iron, haemoglobin, MCV, reticulocytes or transferrin saturation (Tables 2 and S2). In the iron group, ferritin levels increased by 20.4 and 10.6 times at days 14 and 28, respectively.

**Table 2:** Changes in morphometric and biologic characteristics, randomized phase of the study.

	Iron (*n* = 16)			Placebo (*n* = 16)			Effect of IV iron	*P*-value
	Baseline	D14	D28	Baseline	D14	D28		
Morphometry								
Weight, kg	62.1 ± 7.5	61.9 ± 7.5	61.9 ± 7.5	62.9 ± 7.4	62.8 ± 7.6	62.9 ± 7.2	−0.8 [−5.9; 4.4]	0.771
Fat Mass, kg	19.6 ± 9.1	NA	19.6 ± 9.6	18.7 ± 5.5	NA	19.8 ± 7.5	0.9 [−4.4; 6.1]	0.749
Biology								
Iron, µmol/L	15.9 ± 5.0	19.3 ± 6.3	20.3 ± 5.6	17.5 ± 7.0	10.7 ± 4.9	15.5 ± 7.8	0.7 [−3.5; 4.9]	0.738
Ferritin, µg/L	25 ± 12	442 ± 119	232 ± 66	16 ± 8	14 ± 7	14 ± 5	110 [34; 185]	0.004*
Transferrin, µmol/L	31 ± 4	27 ± 4	24 ± 3	33 ± 4	36 ± 5	34 ± 6	−3.2 [−6.4; −0.1]	0.044*
Transferrin saturation, *n* (%^a^)	26 ± 10	36 ± 12	42 ± 13	27 ± 11	15 ± 7	23 ± 10	3.3 [−4.0; 10.6]	0.371
Haemoglobin, g/L	126 ± 9	129 ± 8	125 ± 7	123 ± 8	128 ± 9	119 ± 9	2.0 [−3.8; 7.8]	0.493
MCV, fL	87 ± 4	88 ± 4	89 ± 4	85 ± 3	85 ± 3	85 ± 3	2.3 [−0.3; 4.9]	0.083
Reticulocytes, G/L	51 ± 13	94 ± 18	70 ± 16	51 ± 17	55 ± 24	57 ± 19	9.8 [−4.2; 23.9]	0.170
Reticulocytes, *n* (‰)	12 ± 3	22 ± 5	17 ± 4	12 ± 4	15 ± 8	14 ± 4	2.0 [−1.6; 5.7]	0.275
High-sensitive C-reactive Protein, mg/L	0.91 ± 1.09	0.89 ± 1.16	1.13 ± 1.61	1.07 ± 1.29	1.05 ± 1.08	1.03 ± 0.83	−0.2 [−1.0; 0.6]	0.630

Data are mean ± SD. NA denotes Not Applicable; MCV, Mean Corpuscular Volume and fL, femtoliters.

a The percent transferrin saturation was calculated as iron (in micromoles per liter) ÷ transferrin (in grams per liter) x 25.1. ^£^ Longitudinal changes in biomarkers were analysed using a mixed model for repeated measures adjusted for time. *P* values are for the global effect of the intervention over the 28 days period.

⁎ *P* values reflecting significant results (*P*<0.05).

**Table 3:** Outcomes for the total population, randomized phase of the study.

	Iron (*n* = 15)			Placebo (*n* = 16)			(D28 – baseline) difference between iron and placebo groups [95% CI]; *P* Value
	Baseline	D28	D28 – baseline [95%CI]	Baseline	D28	D28 – baseline [95%CI]	
Primary outcome							
Insulin secretion, iAUC							
First-phase, µU 10 min mL^−1^	86 ± 13	93 ± 14	8 [−13, 29]	76 ± 8	83 ± 9	8 [0, 17]	0 [- 22, 22], *P* = 0.99
Second-phase, first plateau, µU 30 min mL^−1^	523 ± 71	532 ± 94	1 [- 154, 156]	423 ± 44	429 ± 39	6 [- 36, 48]	- 5 [- 161, 151], *P* = 0.95
Second-phase, second plateau, µU 30 min mL^−1^	1755 ± 257	1671 ± 263	- 150 [- 480, 179]	1250 ± 117	1348 ± 182	98 [- 125, 321]	- 249 [- 635, 137], *P* = 0.20
Day of menstrual cycle at which the clamp is performed£, *n*	3.7 ± 2.8	4.1 ± 3.8	0.4 [- 1.3, 2.1]	4.3 ± 3.2	6.3 ± 4.5	2 [0.8, 3.2]	- 1.6 [- 3.7, 0.6], *P* = 0.14
In “first ten days” target menstrual cycle period£, n (%)	15/16 (94)	14/16 (88)	- 6 [- 29, 16]	15/16 (94)	14/16 (88)	- 7 [- 20, 7]	4 [- 26, 27], *P* = 0.97

Data are mean ± SEM or numbers (%). AUC denotes Area Under the Curve.

£ For theses variables, data was available for all the participants of the iron group, even for the participant that refused the follow-up clamp. *P* values are for the effect of the intervention at 28 days.

## Discussion

Herein, we measured changes induced by i.v. iron repletion on glucose homeostasis in iron-deficient, non-anaemic participants using a state-of-the-art method to measure alterations in insulin secretion. Despite acutely increasing and maintaining ferritin values in the “overload” range (ferritin levels > 200 ng/ml in women^1^), the study failed to show any difference regarding first or second phases insulin secretion at 28 days, suggesting no impairment of beta cell function by iron in the chosen setting.

The mechanisms underlying iron's diabetogenic effect are incompletely understood and in part attributable to its free reduced form, known to be particularly toxic for metabolically highly active cells such as beta cells.^1^ Free reduced iron has been shown to increase the production of ROS, that cause oxidative stress, mitochondrial and DNA damage, lipid peroxidation, and protein modification collectively leading to beta cell dysfunction.^1^ It is also unclear, whether iron itself has a proinflammatory effect or whether systemic low-grade inflammation sensitizes beta cells towards iron's toxic effects or both.^1^^,^^6^^,^^7^ Importantly, some minimal levels of iron and ROS being important for normal beta cell function, iron might only become toxic upon further insults such as low-grade inflammation in adipose tissue, or a glucolipotoxic environment.^31^, ^32^, ^33^ In our study, iron induced a decrease in ascorbate levels and an increase in the ascorbyl/ascorbate ratio at day 14 (i.e. suggesting increased ROS production), followed by a return to a physiological anti-oxidant status by day 28. Note that although the total plasma antioxidant capacity (ORAC) was not modified, ascorbate is also a very potent endogenous antioxidant, present in hydrophilic compartments of the human body such as the plasma and extracellular fluids or in the cell's cytosol. In the blood, the total antioxidant capacity is not only related to ascorbate concentration, but also to the presence of other molecules such as albumin and uric acid, which exert synergistic antioxidant effects. Therefore, ascorbate level is believed to reflect, at least partially, the total antioxidant level.^34^^,^^35^ This was nevertheless not accompanied by any impairment in beta cell secretory capacity, nor were any changes in hsCRP levels measured during that same period.

Our trial has some strength. Besides the design and the use of gold standard methods to assess outcomes, we controlled for several important confounding factors. Firstly, as anaemia and consecutive hypoxia could be confounding factors in our observations, anaemia was an exclusion criterion of our study. Secondly, as the menstrual cycle in women could affect glucose homeostasis, the metabolic investigations have been carried out during the first ten days of the menstrual cycle, with very few tests performed outside of this period. Thirdly, we accounted for circadian regulation of glucose homeostasis by starting all metabolic investigations at 6:30 AM. Fourthly, we controlled for differences in caloric intake before the metabolic investigation by providing a standardized meal, the evening before the test.

Our study has also some limitations: while diabetes is believed to be a late complication of HH in humans, the restoration of iron sufficiency might not mimic the time/dose effect of more prolonged absolute iron overload and as such, not fully recapitulate the physiopathology of the disease. Besides, the sample size was small and the study was potentially underpowered to assess differences among groups in the chosen outcome, especially at the second more variable glycaemic step of the clamp also given, that data was missing for 3 out of 16 s steps for reasons previously reported. Furthermore, the choice of measuring changes within 28 days seemed justified by reports demonstrating changes in glucose homeostasis at 1 to 12 weeks of a single phlebotomy.^10^^,^^13^ There were however no available iron infusion trials that assessed similar outcomes to support this decision. In line with this, the choice of measuring main outcomes at 28 days prevents us from drawing firm conclusions regarding very short-term and transitory, or long-term changes due to iron or repeated administrations of iron. Our results can also possibly not be generalized to men as these were not studied or to iron sufficient individuals at baseline that would be prescribed iron infusions to target higher levels of iron stores as it is for example the case for a subset of hearth failure patients. Also, although ferritin values increased towards the overload range, this might reflect the rapid sequestration of iron into tissues of primarily iron deficient participants, rather than “true overload”. Finally, although the use of glucometers to measure blood glucose on arterialized venous blood in clamp studies is increasing and was shown to yield accurate results and to be a reliable substitute to the more fastidious enzymatic methods, we cannot exclude that this choice had a marginal, albeit symmetrical effect on blood glucose measurements in each group.^36^, ^37^, ^38^

In conclusion, acute i.v. iron infusion to apparently healthy but iron deficient women did not alter insulin secretion at 28 days, despite a transient increase in the levels of circulating reactive oxygen species. The small sample size and the consecutive larger data dispersion along with a lack of previous trials to inform sample size calculation could have created a trend towards the null hypothesis. These data should be confirmed in iron deficient men, in patients displaying iron sufficiency at baseline and at other time-points of exposure.

## Data sharing statement

The datasets analysed during the current study are available from the corresponding author on reasonable request.

## Funding

This study was funded by the Swiss National Science Foundation (Grant No 32003B_173092 to Gérard Waeber) and by the University of Lausanne (Young investigator award “Pépinière”), the Leenaards foundation (Junior Clinical Scientist award) and prizes from the Raymond-Berger and Professeur Placide Nicod foundations to Evrim Jaccard.

## Contributors

All authors contributed to the design and conduct of the study and the interpretation of the data. EJ, KS, AG, LB, CG performed the experiments. EJ and PMV had full access to data and analysed the data. EJ, PMV and GW wrote the first draft of the manuscript. All authors provided input into subsequent drafts and approved the final version for submission.

## Declaration of interests

LT has received speaker's fees from Soremartec Italy srl and from Nestlé AG, Switzerland for lectures unrelated to this study.

Other authors have nothing to declare.

## Appendix Supplementary materials

Image, application 1

Image, application 2

Image, application 3

Image, application 4
