# Supplementary material for: Effect of acute iron infusion on insulin secretion: A randomized, double-blind, placebo-controlled trial
Source: eClinicalMedicine. 2022 May 6;48:101434. doi: 10.1016/j.eclinm.2022.101434 (PMC9092517; doi:10.1016/j.eclinm.2022.101434)

**Supplementary material**

**Affiliation of individuals cited in the Acknowledgement section**

Christiane Pellet^1^, Philippe Schneiter^2^, Nathalie Stefanoni^2^, Valentine Rey-Roethlisberger^2^, Aline Voidey^3^, Isabelle Sommer^3^, Julia Lacheze^4^

^1^ Clinical Research Center, CHUV, University of Lausanne, Switzerland.

^2^ Department of Biomedical Sciences, Faculty of Biology and Medicine, University of Lausanne, rue du Bugnon 7a, 1005 Lausanne, Switzerland.

^3^ Pharmacy Unit, Department of interdisciplinary Centres, Lausanne University Hospital (CHUV), rue du Bugnon 46, 1011 Lausanne, Switzerland.

^4^ Research team, Pathophysiology and Epidemiology of Cerebro-Cardiovascular diseases (PEC2, EA7460), University of Bourgogne Franche-Comté, UFR des Sciences de Santé, 7 boulevard Jeanne d’ Arc, 21079 DIJON, France

**Supplementary tables**

**Table S1. Baseline Characteristics of the Eligible and Non-Eligible Population**

| **Characteristics** | | **Eligible (n=32)** | **Non-Eligible (n=40)** |
| --- | --- | --- | --- |
| Age, years | | 27.8 ± 7.3 | 32.3 ± 8.9 |
| Ethnicity, n (% Caucasian^a^) | | 75 | 80 |
| Body weight, kg | | 62.5 ± 7.3 | 64.1 ± 8.7 |
| BMI, kg/m^2^ | | 22.6 ± 2.4 | 23.5 ± 2.9 |
| Heart rate, beats per min | | 65 ± 8 | 65 ± 9 |
| Systolic BP, mm Hg | | 110 ± 10 | 109 ± 10 |
| Diastolic BP, mm Hg | | 71 ± 9 | 72 ± 7 |
| Waist circumference, cm | | 71 ± 6 | 75 ± 9.0 |
| Hip circumference, cm | | 100 ± 7 | 100 ± 10 |
| Waist-Hip ratio, *n* | | 0.71 ± 0.05 | 0.76 ± 0.17 |
| VAS of fatigue, *n* | | 6.34 ± 1.25 | 6.23 ± 1.25 |
| PHQ-2, *n* | | 1.1 ± 1.0 | 1.2 ± 1.0 |
| Iron perfusions in the past, *n* | | 0.5 ± 1.5 | 1.0 ± 29.0 |
| Hormonal contraception, n (%) | | 8/32 (25) | 11/40 (28) |
| Laboratory measurements | |  |  |
|  | Haemoglobin, g/L | 133 ± 8 | 130 ± 9 |
|  | MCV, fL | 86 ± 4 | 87 ± 4 |
|  | Serum ferritin, µg/L | 21 ± 8 | 67 ± 69 |
|  | Transferrin saturation, *n* (%^b^) | 0.22 ± 0.09 | 0.24 ± 0.10 |
|  | C-reactive protein, mg/L | 2.2 ± 1.2 | 2.0 ± 2.2 |
|  | Venous glycaemia, mmol/L | 4.8 ± 0.4 | 4.7 ± 0.5 |
|  | HbA_1c_, *n* (%) | 5.0 ± 0.3 | 4.9 ± 0.4 |
|  | Thyroid stimulating hormone, mUI/L | 2.23 ± 1.18 | 2.91 ± 0.88 |
|  | Vitamin B9, nmol/L | 18.6 ± 7.5 | 16.7 ± 7.6 |
|  | Vitamin B12, pmol/L | 262 ± 86 | 334 ± 137 |
|  | Alanine aminotransferase, U/L | 15 ± 4 | 18 ± 6 |
|  | Aspartate aminotransferase, U/L | 18 ± 4 | 19 ± 5 |

Data are mean ± SD, numbers (%).BMI denotes Body Mass Index; BP, Blood Pressure; VAS, Visual Analogue Scale; MCV, Mean Corpuscular Volume; fL, femtoliters and PHQ-2, Patient Health Questionnnaire-2.

^a^ Ethnicity was self-reported.

^b^ The percent transferrin saturation was calculated as iron (in micromoles per liter) ÷ transferrin (in grams per liter) x 25.1.

**Table S2. Morphometric, clinical and biologic characteristics, with extension arm**

|  | **Iron (n = 19)** | **Placebo (n = 16)** | **Effect of IV iron** | ***P*-value** |
| --- | --- | --- | --- | --- |
|  | **D28 + ext^a^** | **D28** |  |  |
| Morphometry |  |  |  |  |
| Weight, kg | 61.5 ± 7.1 | 62.9 ± 7.2 | -0.5 [-1.6 ; 0.5] | P=0.333 |
| Biology |  |  |  |  |
| Iron, µmol/L | 20.3 ± 5.4 | 15.5 ± 7.8 | 0.6 [-3.5 ; 4.7] | P=0.777 |
| Ferritin, µg/L | 227 ± 65 | 14 ± 5 | 113 [38 ; 188] | P=0.003 |
| Transferrin, µmol/L | 25 ± 3 | 34 ± 6 | -2 [-5; 0] | P=0.040 |
| Transferrin saturation, *n* (%^b^) | 40 ± 13 | 23 ± 10 | 2.6 [-4.5 ; 9.7] | P=0.478 |
| Haemoglobin, g/L | 124 ± 7 | 119 ± 9 | 2 [-4; 7] | P=0.560 |
| MCV, fL | 88 ± 4 | 85 ± 3 | 0.8 [- 0.8 ; 2.4] | P=0.313 |
| Reticulocytes, G/L | 69 ± 15 | 57 ± 19 | 8 [-6 ; 21] | P=0.257 |
| Reticulocytes, n (‰) | 17 ± 4 | 14 ± 4 | 2 [-2 ; 5] | P=0.333 |
| Ultrasensitive C-reactive Protein, mg/L | 1.03 ± 1.50 | 1.03 ± 0.83 | -0.4 [-1.0 ; 0.3] | P=0.225 |

Data are mean ± SD. MCV denotes Mean Corpuscular Volume and fL, femtoliters.

^a^ ext = extension: pooled post-baseline results including extension arm participants.

^b^ The percent transferrin saturation was calculated as iron (in micromoles per liter) ÷ transferrin (in grams per liter) x 25.1.

^£^ Longitudinal changes in biomarkers were analysed using a mixed model for repeated measures adjusted for time. *P* values are for the global effect of the intervention over the 28 days period.

| **Table S3. Outcomes for the total population, with extension arm** | | | | | | | |
| --- | --- | --- | --- | --- | --- | --- | --- |
|  | **Iron (n=18)** | | | **Placebo (n=16)** | | | **(D28 – baseline) difference between iron and placebo groups [95% CI]; *P* Value** |
|  | **Baseline** | **D28** | **D28 – baseline [95%CI]** | **Baseline** | **D28** | **D28 – baseline [95%CI]** |  |
| Primary outcome | | | | | | | |
| Insulin secretion, iAUC | | | | | | | |
| First-phase, µU 10min mL^-1^ | 85 ± 11 | 93 ± 12 | 8 [- 10, 26] | 76 ± 8 | 84 ± 9 | 8 [0, 16] | 0 [-20, 20], *P*=0.99 |
| Second-phase, first plateau, µU 30min m^-1^L | 523 ± 61 | 549 ± 80 | 20 [- 111, 148] | 423 ± 44 | 429 ± 39 | 6 [- 36, 48] | 13 [- 130, 157], *P*=0.85 |
| Second-phase, second plateau, µU 30min mL^-1^ | 1713 ± 218 | 1661 ± 221 | - 150 [- 480, 179] | 1250 ± 117 | 1348 ± 182 | 98 [-125, 321] | - 200 [- 550, 149], *P*=0.25 |
| Day of menstrual cycle at which the clamp is performed^£^, *n* | 4.3 ± 0.7 | 4.6 ± 1.0 | 0.3 [-1.3, 2.0] | 4.3 ± 0.8 | 6.3 ± 1.1 | 2 [0.8, 3.2] | - 1.7 [-3.8, 0.5], *P*=0.12 |
| In “first ten days” target menstrual cycle period^£^, n (%) | 18/19 (95) | 16/19 (84) | - 10 [- 32, 11] | 15/16 (94) | 14/16 (88) | - 7 [- 20, 7] | - 4 [- 30, 23], *P*=0.77 |
| Data are mean ± SEM or numbers (%).AUC denotes Area Under the Curve.  *P* values are for the effect of the intervention at 28 days  ^£^ For theses variables, data was available for all the participants of the iron group, even for the participant that refused the follow-up clamp.  ^a^ ext = extension: pooled post-baseline results including extension arm participants. | | | | | | | |

**Supplementary figure legends**

**Figure S1 | Study timeline.** We planned each hyperglycaemic clamp (visit V2 and V5) during the presumed first 10 days of the menstrual cycle of the participant. Each of the clamps was preceded the evening before, by a standardized meal adapted to energy requirements. We performed the baseline hyperglycaemic clamp of visit V2 within seven days of visit V3 (usually the same day). Unblinded study staff thereafter ensured study drug administration at V3 and participant follow-up. Visit V4 and V5 were scheduled 14 ± 2 and 28 ± 2 days after V3 respectively. The investigators performed the hyperglycaemic clamps of visit V2 and V5 but had no contact with participants and no access to their files during the randomized period of the trial. The extension arm of the study was optional.

**Figure S2 | Timeline of the two-step hyperglycaemic clamp**. At -150 min, a primed 2 mg/kg continuous 20 μg/kg/min infusion of 6,6^2^H_2_ glucose was started on one of the venous cannula and continued throughout the basal period of the test (time -150 to 0 min). Thereafter, a primed variable infusion of exogenous glucose 20% labelled with 1.25% 6,6^2^H_2_ glucose was started. It allowed to increase plasma glucose levels to reach a 1^st^ plateau at 7.5 mmol/L glucose that was maintained for 60 min (time 30 to 90 min) and then to reach a 2^nd^ plateau at 10 mmol/L glucose that was maintained for an additional 60 min (time 120 to 180 min). Blood glucose was measured by a glucometer, on arterialized venous blood drawn from the opposite forearm catheter, at indicated times (red dots). Blood was also drawn (red arrows), immediately centrifuged and plasma/serum were aliquoted and stored at -20°C until analysed. The 1^st^ phase insulin secretion (IS) was assessed during the first 0 to 10 min of the clamp. The 2^nd^ phase IS was assessed during the second one-half of each plateau (time 60 to 90 min and 150 to 180 min) when blood glucose levels are at steady state.

**Supplementary figures**

**Figure S1**


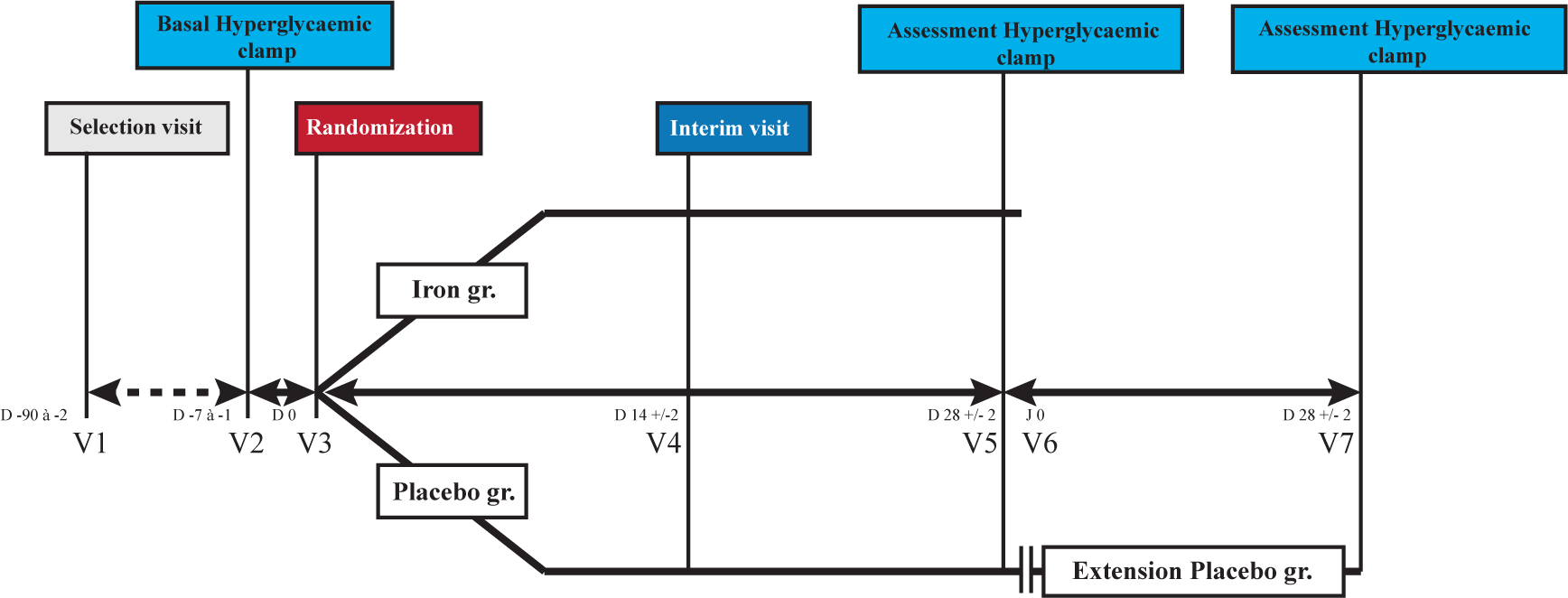


**Figure S2**


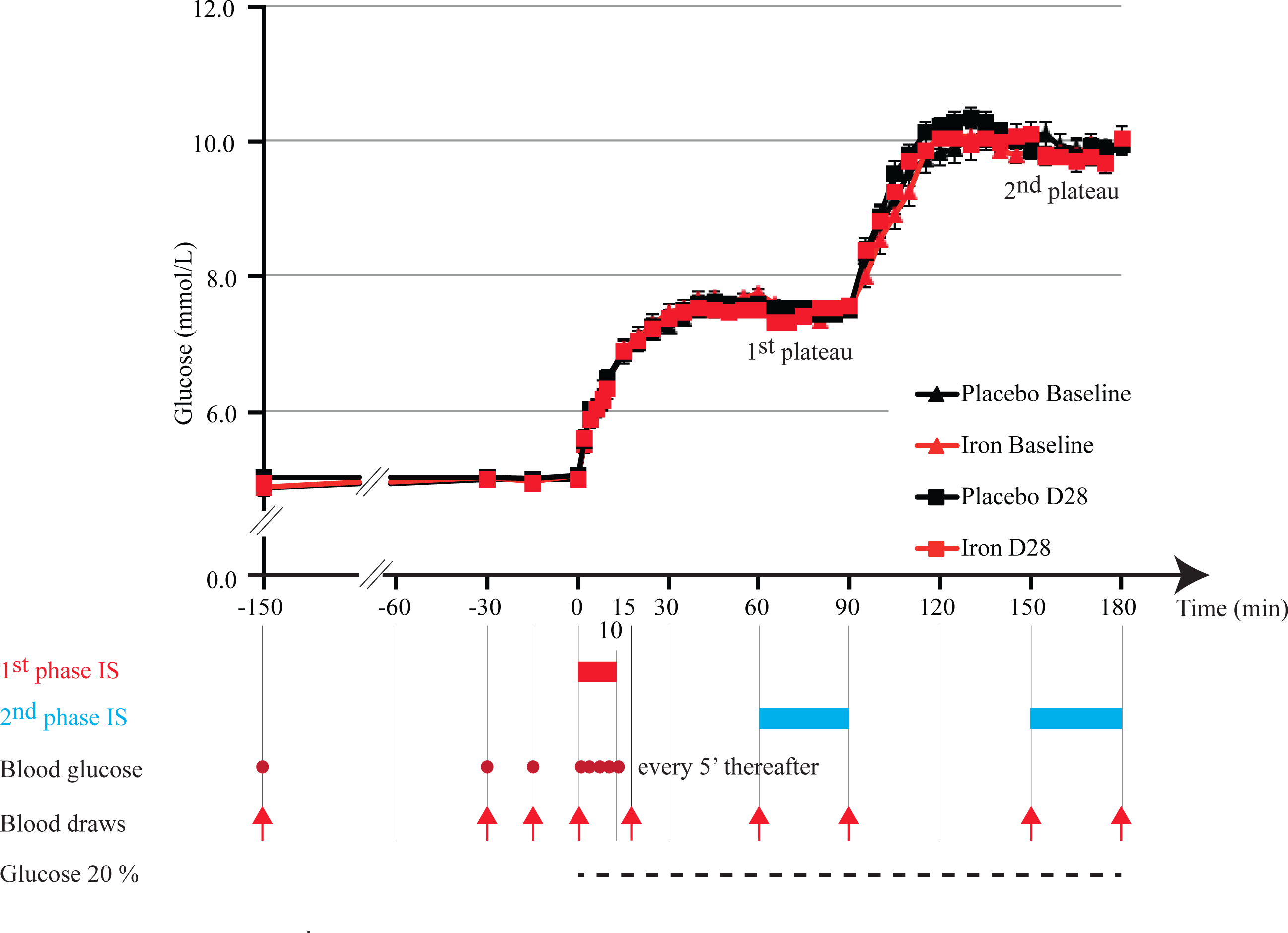

Supplement: Supplementary file 1 [file mmc1.docx]
