# Supplementary material for: Effect of acute iron infusion on insulin secretion: A randomized, double-blind, placebo-controlled trial
Source: eClinicalMedicine. 2022 May 6;48:101434. doi: 10.1016/j.eclinm.2022.101434 (PMC9092517; doi:10.1016/j.eclinm.2022.101434)

**Supplementary Figure 1: Study timeline**


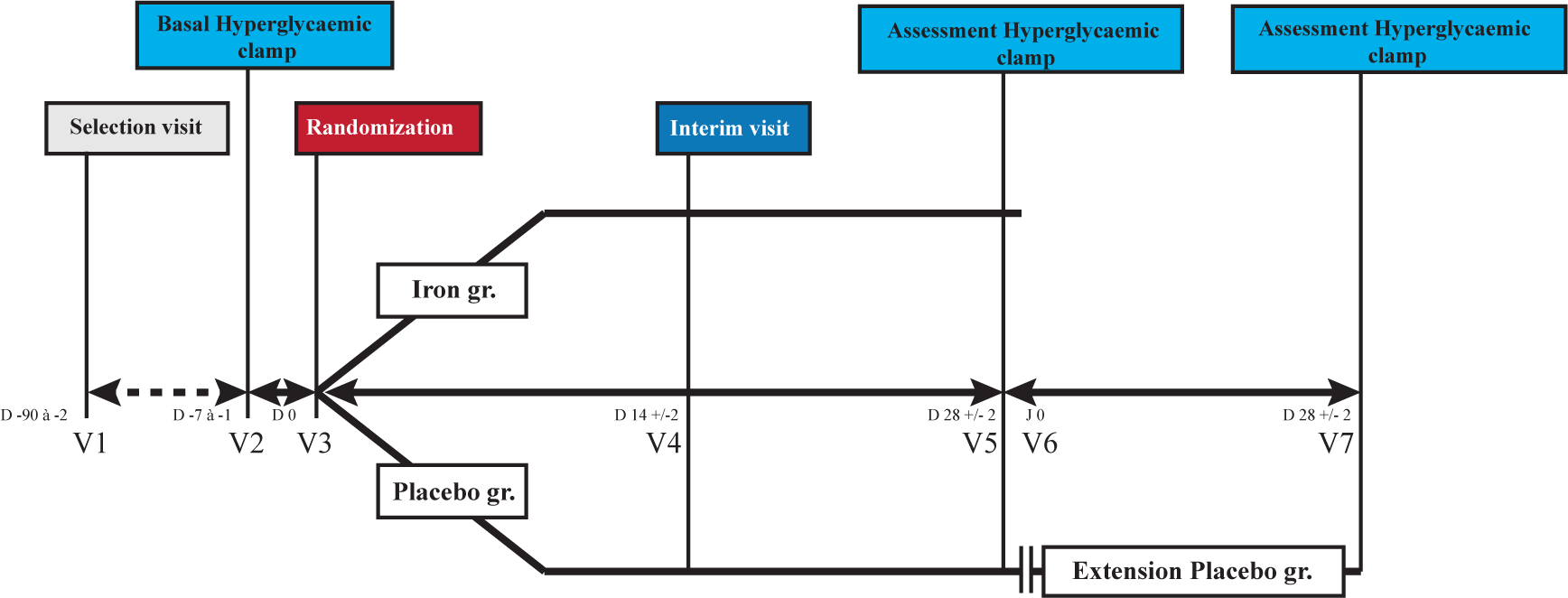


**Supplementary Figure 2: Timeline of the two-step hyperglycaemic clamp**


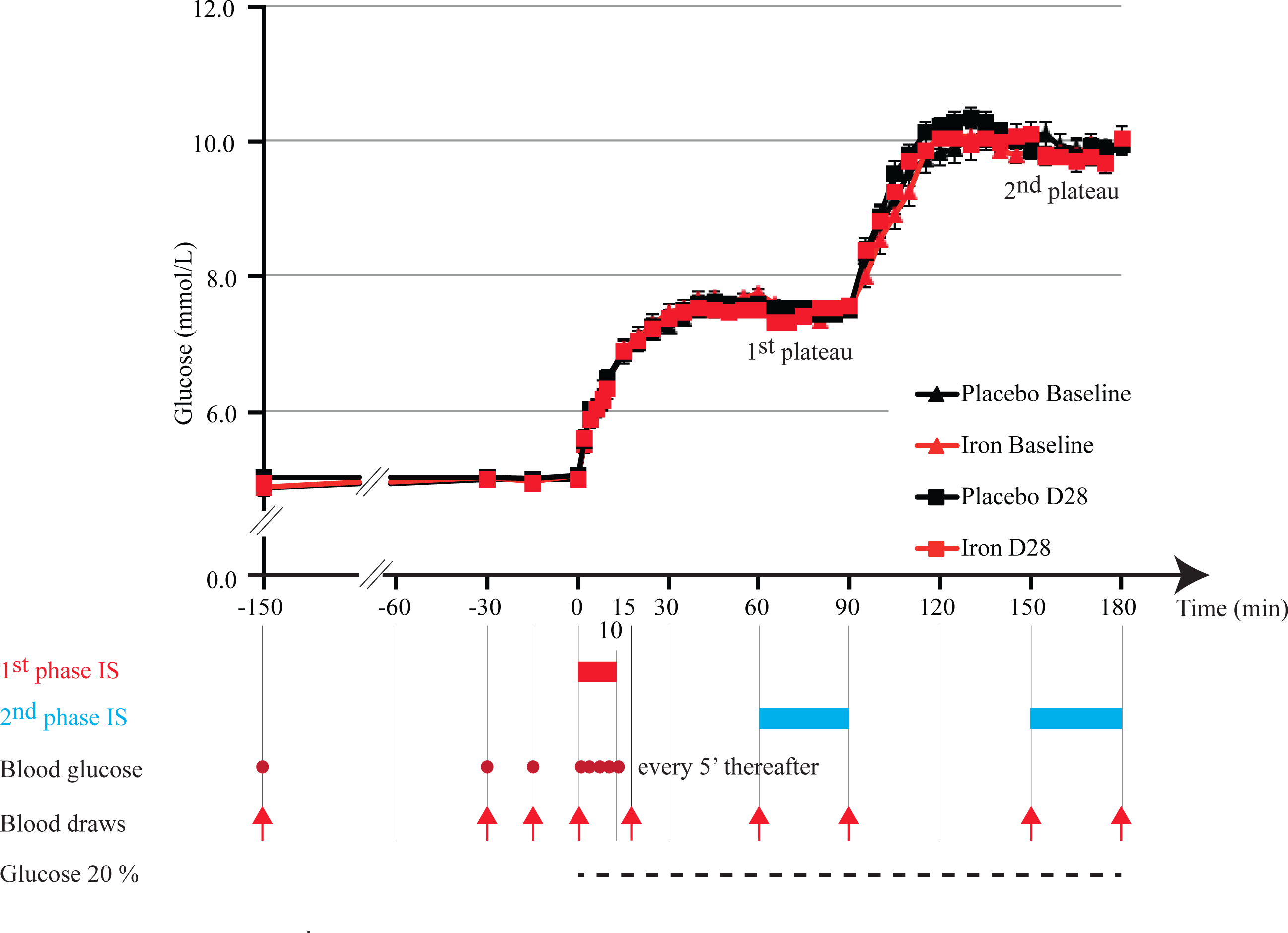

Supplement: Supplementary file 3 [file mmc3.docx]
