# Supplementary figures and images for: Effect of acute iron infusion on insulin secretion: A randomized, double-blind, placebo-controlled trial
Source: eClinicalMedicine. 2022 May 6;48:101434. doi: 10.1016/j.eclinm.2022.101434 (PMC9092517; doi:10.1016/j.eclinm.2022.101434)

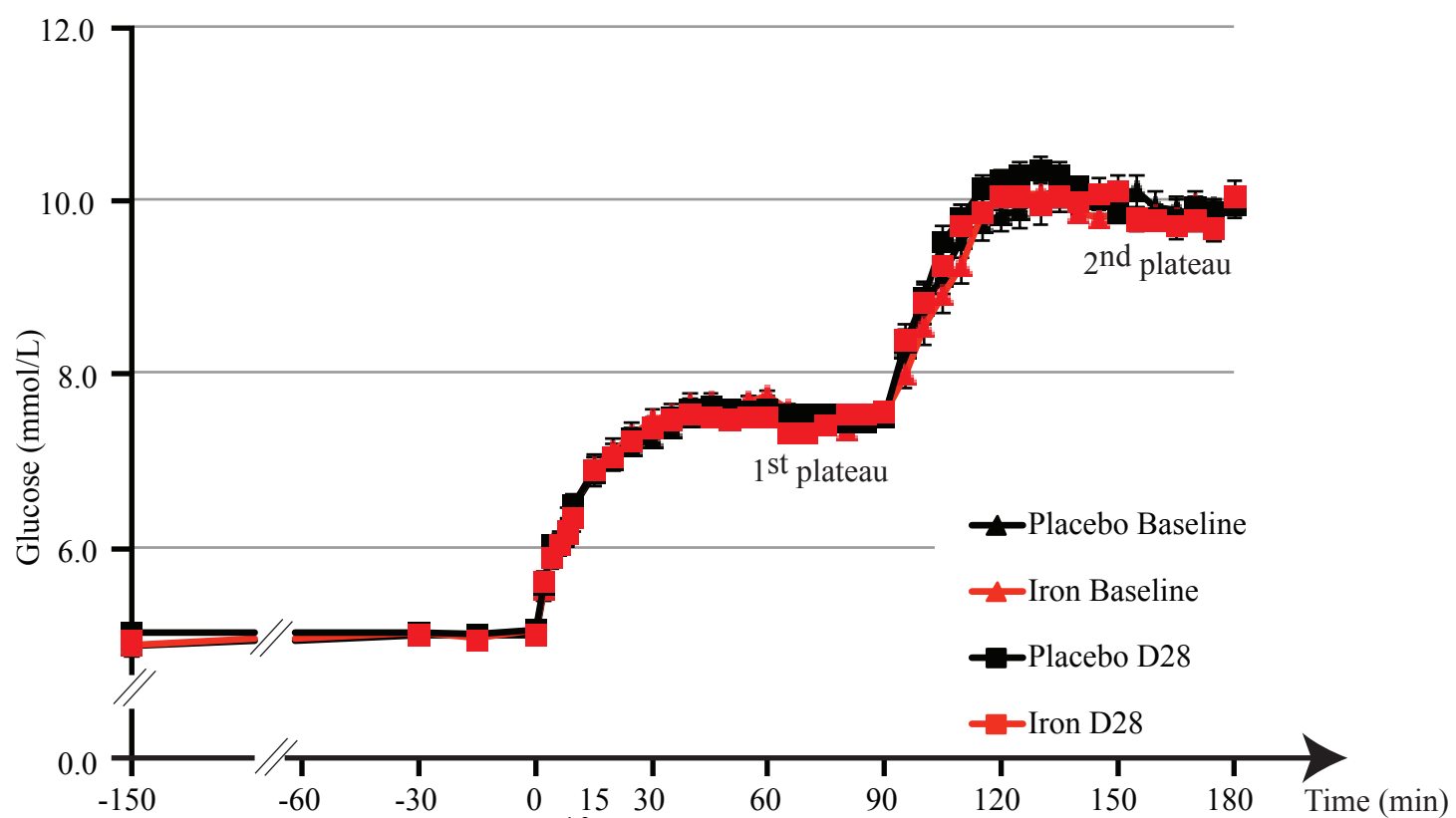

1<sup>st</sup> phase IS

2<sup>nd</sup> phase IS

Blood glucose

Blood draws

Glucose 20 %

10  
every 5' thereafter

Supplement: Supplementary file 4 [file mmc4.pdf]
